# Supplementary material for: Cation desolvation-induced capacitance enhancement in reduced graphene oxide (rGO)
Source: Nat Commun. 2024 Mar 2;15:1935. doi: 10.1038/s41467-024-46280-1 (PMC10908864; doi:10.1038/s41467-024-46280-1)
Supplement: Supplementary file 1 — Supplementary Information [file 41467_2024_46280_MOESM1_ESM.pdf]

Table of contents

Supplementary Figure 1. Temperature-programmed desorption mass spectrometry (TPD-MS) of rGO

Supplementary Figure 2. Cation-dependent cyclic voltammetry curves of rGO

Supplementary Figure 3. Cyclic voltammetry curves at different scan rates and kinetic analysis of rGO

Supplementary Figure 4. Cyclic voltammetry curves of rGO and r^2GO

Supplementary Figure 5. Validation of gravimetrical model of EQCM measurements

Supplementary Figure 6. XRD patterns of rGO electrodes

Supplementary Figure 7. Thickness measurements of rGO electrodes

Supplementary Figure 8. EQCM calibration plot of the coating in air (frequency vs. mass for different loadings)

Supplementary Table 1. Integrated capacitance of rGO and r^2GO in different electrolytes

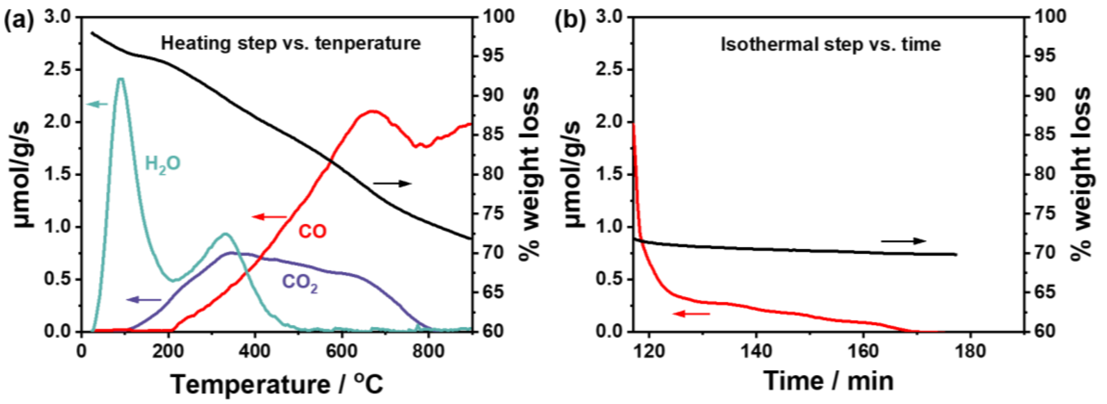

(c)

	H_2O µmol/g	H_2O wt %	CO µmol/g	CO wt %	CO_2 µmol/g	CO_2 wt%	O wt%
rGO	1827	3.3	6581	18.4	1934	8.5	16.7
rGO Thermal treatment Heating step	1824	3.3	5220	15.4	1958	8.6	14.6
rGO Thermal treatment Isothermal step	-	-	727	2.0	-	-	1.2
rGO after thermal treatment (r^2GO)	304	0.5	801	2.2	149	0.7	1.3

Supplementary Figure 1. Temperature-programmed desorption mass spectrometry (TPD-MS) of rGO. Weight loss in % and gas evolution in µmol/g/s after quantification for H_2O, CO, CO_2, and arbitrary units for the other gases during **(a)** heating step and **(b)** isothermal step. The content of surface oxygen is 16.7 wt.% of rGO and 1.3 wt.% of r^2GO as shown in **(c)**.

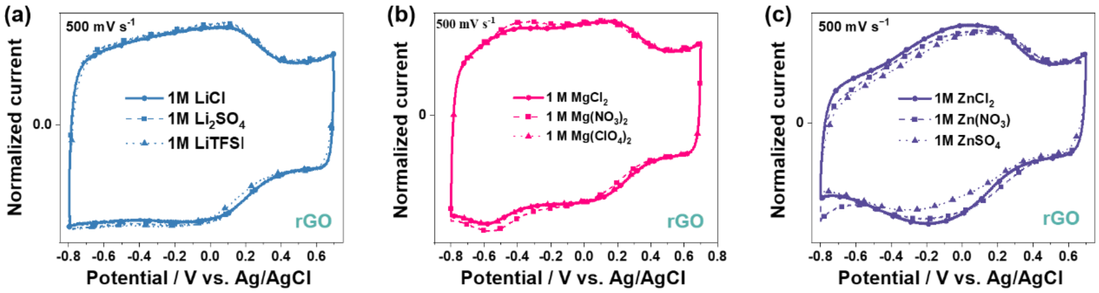

Supplementary Figure 2. Cation-dependent cyclic voltammetry curves of rGO. Cyclic voltammetry curves of rGO when applying different electrolytes varying in anions while the cations of **(a)** Li^+, **(b)** Mg^{2+}, and **(c)** Zn^{2+} remain fixed, in three-electrode cavity micro-electrode setup with the scan rate of 500 mV s^{-1}.

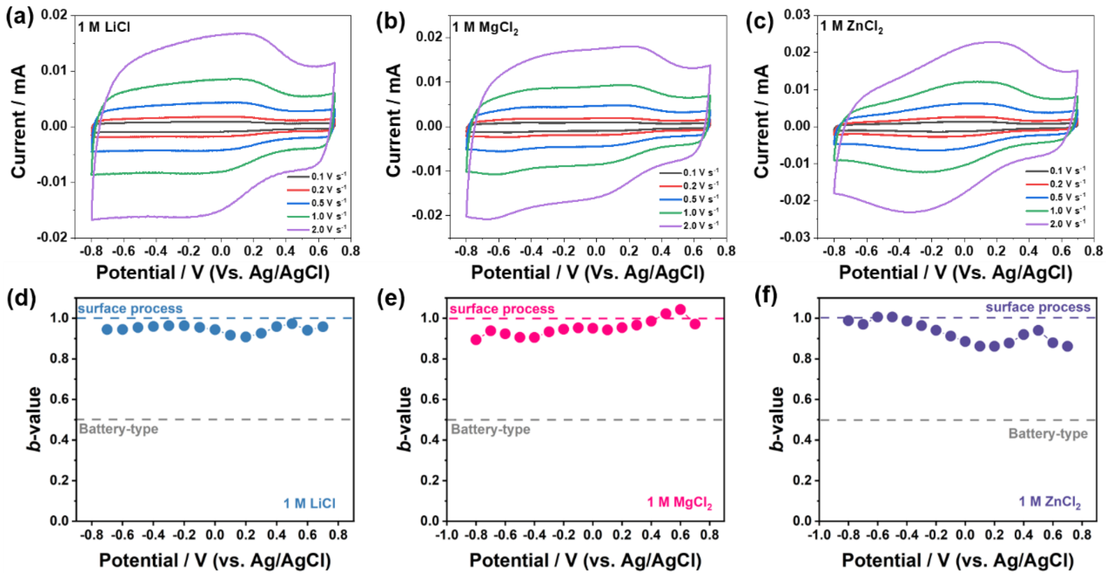

Supplementary Figure 3. Cyclic voltammetry curves at different scan rates and kinetic analysis of rGO. (a-c) Cyclic voltammetry curves at the scan rates of 0.1, 0.2, 0.5, 1.0, and 2.0 V s^{-1} of rGO. **(d-e)** the *b*-value obtained by linear fitting of the logarithm of the cathodic bump/peak current against the scan rate at different potentials, when applying different electrolytes in three-electrode cavity micro-electrode configuration. The electrolytes are (a) (d) 1 M LiCl; (b) (e) 1 M $MgCl_2$; and (c) (f) 1 M $ZnCl_2$.

The current response follows the equation $i = a\,v^b$, where a and b are fitting parameters. The b-exponent is expected to land between 0.5 and 1. If the current is limited by the semi-infinite diffusion of the reactive species, $b = 0.5$, for example battery-type electrodes. Differently, b reaches 1 when the current is surface-controlled, i.e. surface process. We plotted the b-values for different potential showing it close to 1, among various electrolytes, suggesting current linear increase with scan rate and confirming that the charge storage is driven by a surface charge storage process without diffusion limitation.

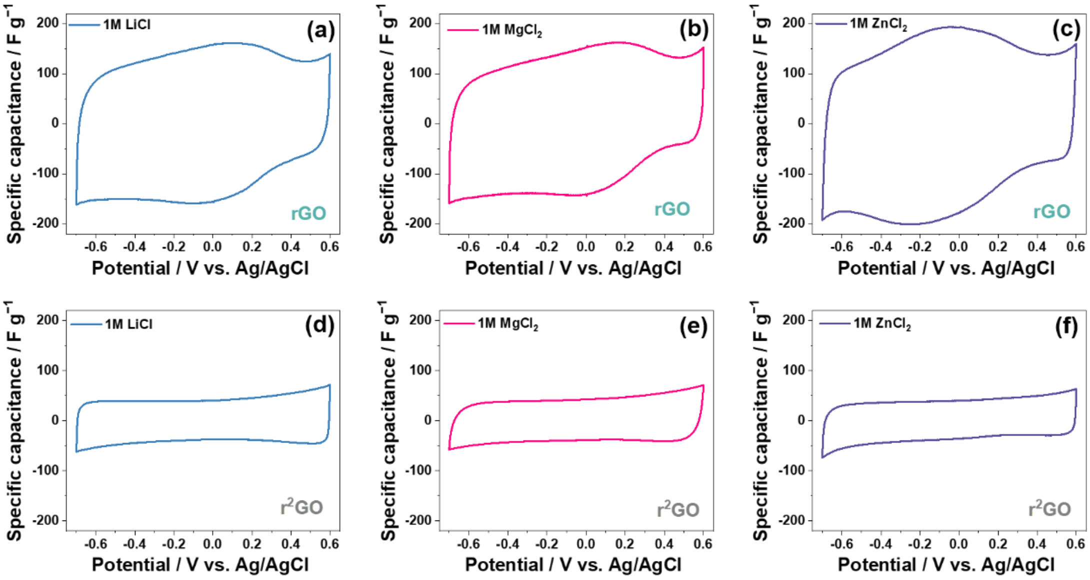

Supplementary Figure 4. Cyclic voltammetry curves of rGO and r^{2}GO. (a-c) cyclic voltammetry curves of rGO, **(d-f)** cyclic voltammetry curves of r^{2}GO at the scan rate of 200 mV s^{-1} when applying different electrolytes in three-electrode Swagelok cells.

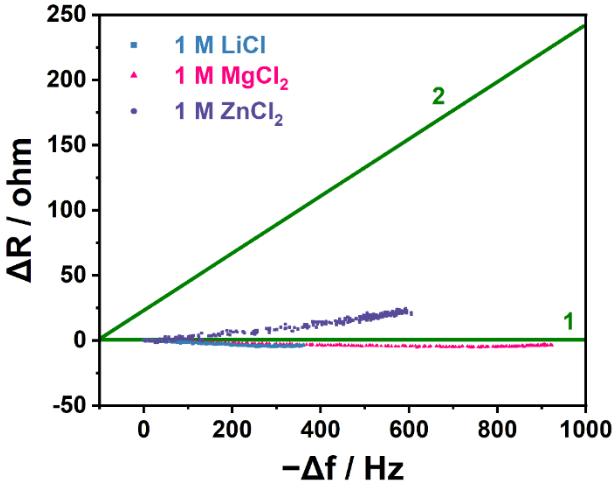

Supplementary Figure 5. Validation of gravimetrical model of EQCM measurements. Diagram of the resonant montional resistance change (ΔR) and resonant frequency change (−Δf) for rGO during the cathodic charging with 1M LiCl, 1M $MgCl_2$, and 1M $ZnCl_2$ electrolyte. Green line 1 and 2 (reproduced from Ref. S1)[S1] represent an elastic mass effect and a pure viscosity–density effect, respectively.

The negligible change of the motional resistance ΔR vs −Δf evidences a rigid behavior of the coating during polarization, thus validating the gravimetric analysis of the electrochemical behavior of the rGO coatings in the different electrolytes.

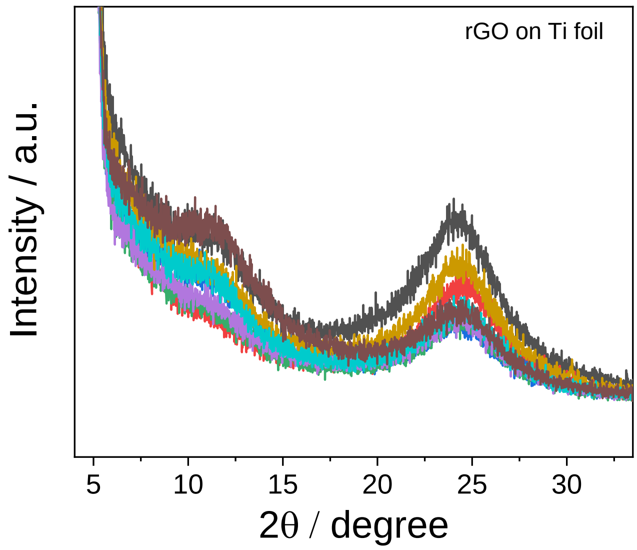

Supplementary Figure 6. XRD patterns of rGO electrodes. The rGO electrodes are made by coating of rGO on titanium current collector. The different plots represent the X-ray diffraction data collected at different positions of the same electrode. Most of the unobvious diffraction peaks at 2θ=11-13° demonstrate the random stacking of rGO particles.

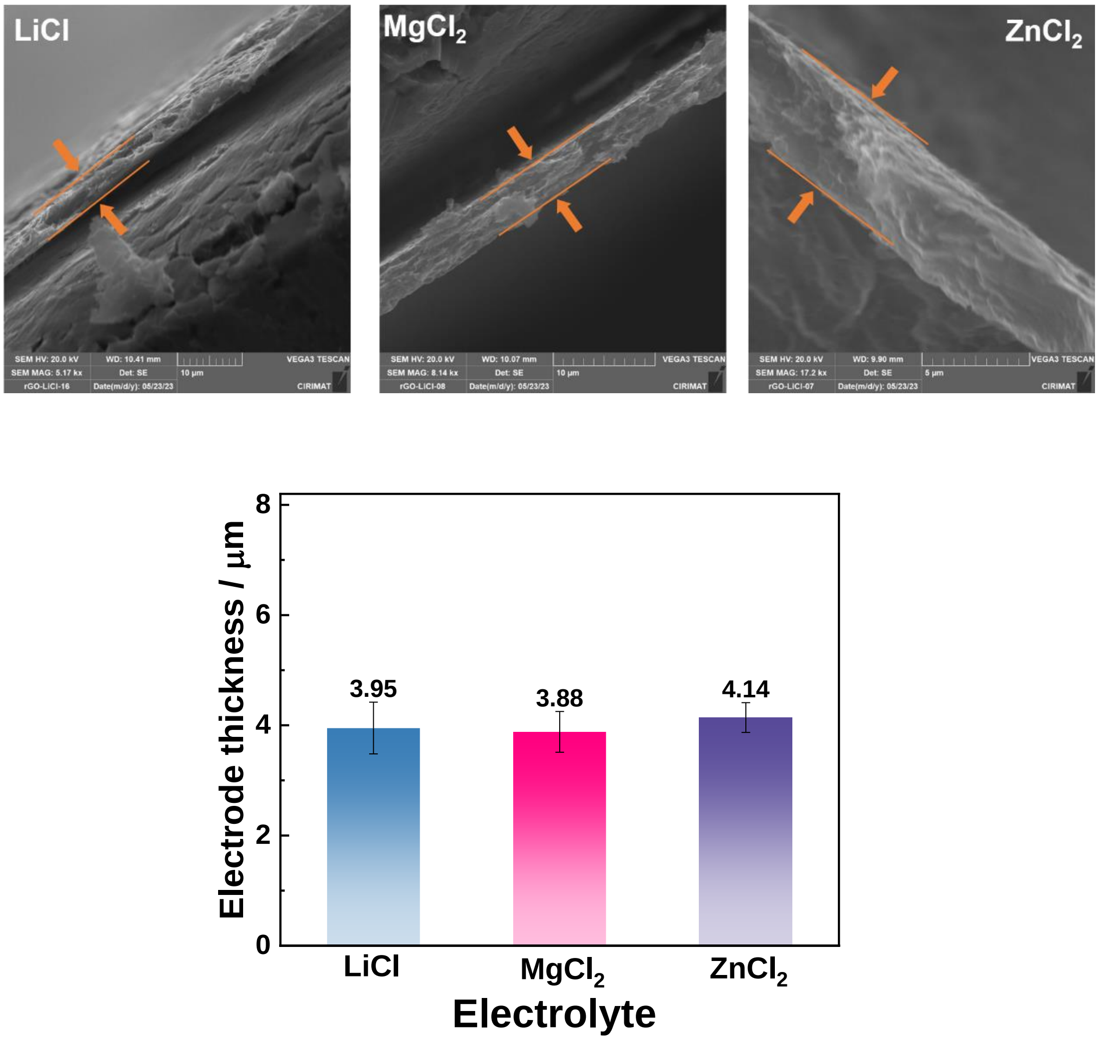

Supplementary Figure 7. Thickness measurements of rGO electrodes. The thickness of rGO electrodes that have been used in in-situ electrochemical dilatometry measurements are determined by scanning electron microscope (SEM). The current collector in the background is Titanium foil. The presented average thickness value, along with error bars, was obtained through multiple measurements taken from various observation frames.

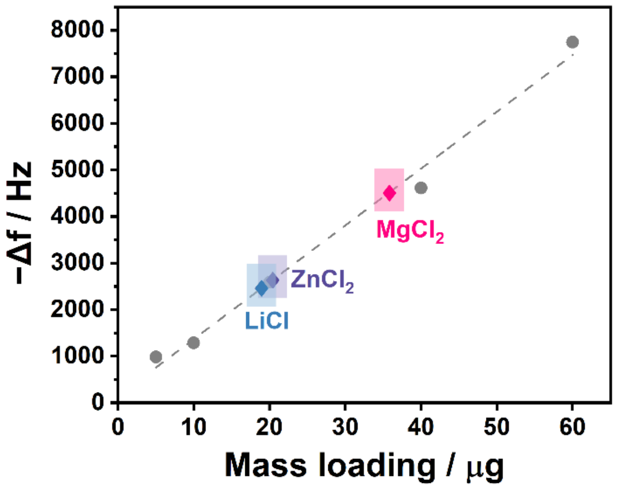

Supplementary Figure 8. EQCM calibration plot of the coating in air (frequency vs. mass for different loadings). Supplementary Figure 8 shows that the rGO weight loading selected for EQCM experiments falls within the linear change of frequency vs. mass, evidencing the presence of a rigid rGO coating in air.

Supplementary Table 1. Integrated capacitance of rGO and r^2GO in different electrolytes (obtained by integrating the CV curves shown in Supplementary Figure 4)

Solution / 1 M	Capacitance of rGO / F g^{-1}	Capacitance of r^2GO / F g^{-1}
LiCl	129	43
$MgCl_2$	121	42
$ZnCl_2$	152	39

Supplementary References

S1. Su X.; Li Y. A QCM Immunosensor for Salmonella Detection with Simultaneous Measurements of Resonant Frequency and Motional Resistance. *Biosens. Bioelectron.* **21**, 840-848 (2005).
